# Total Femoral Head Collapse in a Corticosteroid-Induced Pathologic Fracture

**DOI:** 10.7759/cureus.81543

**Published:** 2025-03-31

**Authors:** Joshua L Dale, Zain Sayeed

**Affiliations:** 1 Osteopathic Medicine, William Carey University College of Osteopathic Medicine, Hattiesburg, USA; 2 Orthopedics, Doctors Hospital at Renaissance, Edinburg, USA

**Keywords:** corticosteroid use, elderly trauma, ficat, surgery, total hip arthoplasty

## Abstract

Glucocorticoid therapy is a well-established iatrogenic cause of secondary osteoporosis. This form of osteoporosis is known as glucocorticoid-induced osteoporosis (GIOP). We present the case of a 78-year-old woman who developed avascular necrosis and complete collapse of the femoral head after a high-dose steroid taper for asthma. Imaging showed subsequent femoral head collapse with the presence of protrusio acetabuli. The patient underwent a posterior total hip arthroplasty. This case is unique because of the extremely high dose of steroids and the timing of her fracture. The case reveals the adverse effects of prolonged glucocorticoid use on bone health, showing the need for proper preventive measures such as bone density scans and proper supplementation if needed. Using anti-osteoporotic medications such as bisphosphonates or denosumab can mitigate the occurrence of GIOP. Early recognition and proper management are critical in decreasing the incidence of GIOP-related fractures.

## Introduction

Glucocorticoid therapy is the leading iatrogenic cause of secondary osteoporosis [1]. Glucocorticoid-induced osteoporosis (GIOP) affects approximately 30-50% of individuals undergoing extended glucocorticoid therapy. This is typically defined as the use of doses equivalent to 5 mg or more of prednisone daily for three months or longer [2]. Research by Lai et al. demonstrated that higher doses of steroids significantly increase the risk of hip fractures, with some studies showing a more than 2.5-fold higher likelihood of fractures in patients using steroids for extended periods, regardless of their age group [3,4].

One of the most common uses of steroid therapy is in the treatment of asthma and other respiratory conditions [5]. However, glucocorticoids affect more than just the inflammatory cascade involved in asthma pathogenesis. They reduce serum levels of P1NP and osteocalcin, both of which are key markers in bone formation. Interestingly, upon discontinuation of corticosteroids, these markers show a reversal of their decline [6].

There is also evidence showing that extended exposure to corticosteroids can predispose patients to avascular necrosis (AVN) in the femoral head. The femoral head is the most common site of AVN in patients receiving corticosteroids, occurring in up to 3-40% of patients who are using them for treatment [7]. Scaling for avascular necrosis is graded using the Ficat classification, which classifies the five different stages of bone necrosis [8]. The scaling would range from stage I, which is the earliest stage, to stage IV, where you would start to witness the femoral head collapse on a pure radiograph [9].

## Case presentation

A 78-year-old woman presented to the emergency department for left hip pain. The patient’s history was pertinent for asthma, for which she took an inhaler whenever she had flares and would use Solu-Medrol dose packs in the past, which were prescribed to her previously in the case of severe flares. The patient denied any history of smoking or drinking as well. The patient reported experiencing hip pain for the past three months, with a marked worsening of symptoms over the last two weeks. She claimed that the initial pain felt similar to the osteoarthritis pain she had in her right hip, which had prompted her to obtain a total hip arthroplasty in the past. The patient denied any history of trauma or falls in recent history upon investigation. She disclosed a history of oral glucocorticoid use over the preceding five weeks. The glucocorticoid regimen consisted of a tapering dosage as follows: four pills daily during the first week, three pills daily during the second week, two pills daily during the third week, one pill daily during the fourth week, and one pill every other day during the fifth week. The exact dosage of prednisone was 5 mg.

On physical examination, the patient demonstrated significant discomfort, with a shortened and externally rotated left lower extremity. Examination of the right hip, which had previously undergone successful replacement, revealed no abnormalities. The sensation was intact bilaterally across both lower extremities, and strength was preserved in the right lower extremity. Labs ordered ruled out any infectious or metastatic cause; results from the day of the patient consult are summarized in Table 1. The patient's anemia was attributed to blood loss from the fracture.

**Table 1:** Patient lab results with reference ranges

Test	Patient Value (Reference Range)
White Blood Cell (WBC)	9.40 (4.0–11.0 x 10^9^/L)
Red Blood Cell (RBC)	3.36 (4.5–5.5 x 10^12^/L)
Hemoglobin	10.0 (13.0–16.0 g/dL)
Hematocrit	33.7 (37–47%)
Platelet Count	207 (150–450 x 10^9^/L)
Absolute Neutrophils	6.84 (1.8–7.7 x 10^9^/L)
Absolute Lymphocytes	1.57 (1.0–4.8 x 10^9^/L)
Absolute Monocytes	0.51 (0.2–0.8 x 10^9^/L)
Absolute Eosinophils	0.20 (0.0–0.5 x 10^9^/L)
Absolute Basophils	- (0.0–0.2 x 10^9^/L)
Absolute Immature Granulocytes	0.21 (0.0–0.03 x 10^9^/L)
Percent Neutrophils	72.9% (40–60%)
Percent Monocytes	5.40% (2–8%)
Percent Eosinophils	2.1% (1–4%)
Percent Basophils	0.7% (0.5–1%)
Percent Immature Granulocytes	2.2% (0–1%)

**Figure 1:**
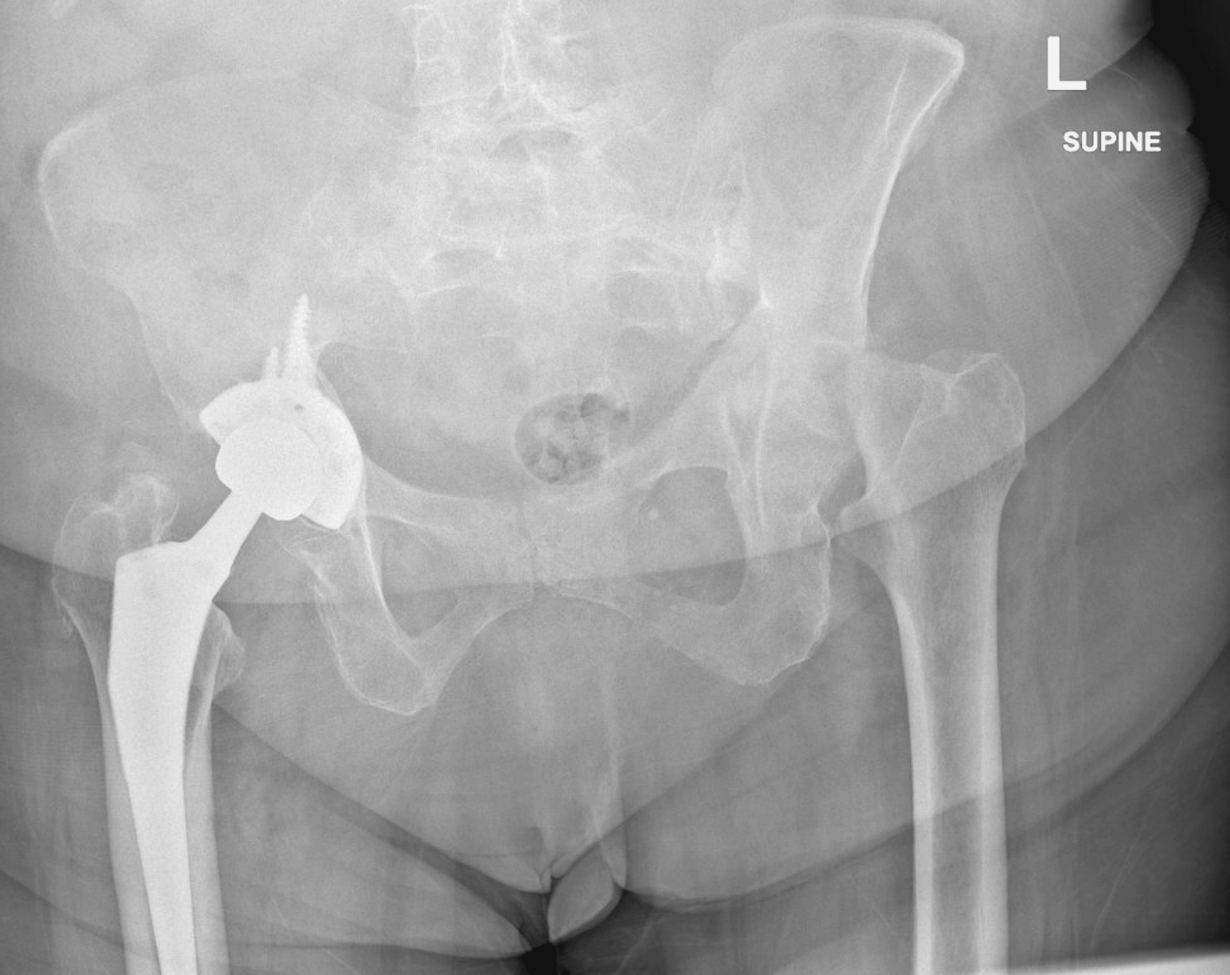
Preoperative AP X-ray of the pelvis AP: anteroposterior

**Figure 2:**
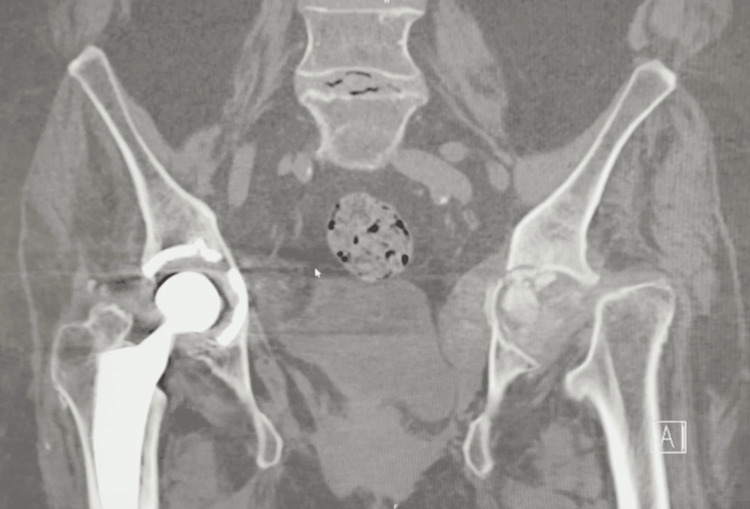
Preoperative coronal CT of the pelvis

**Figure 3:**
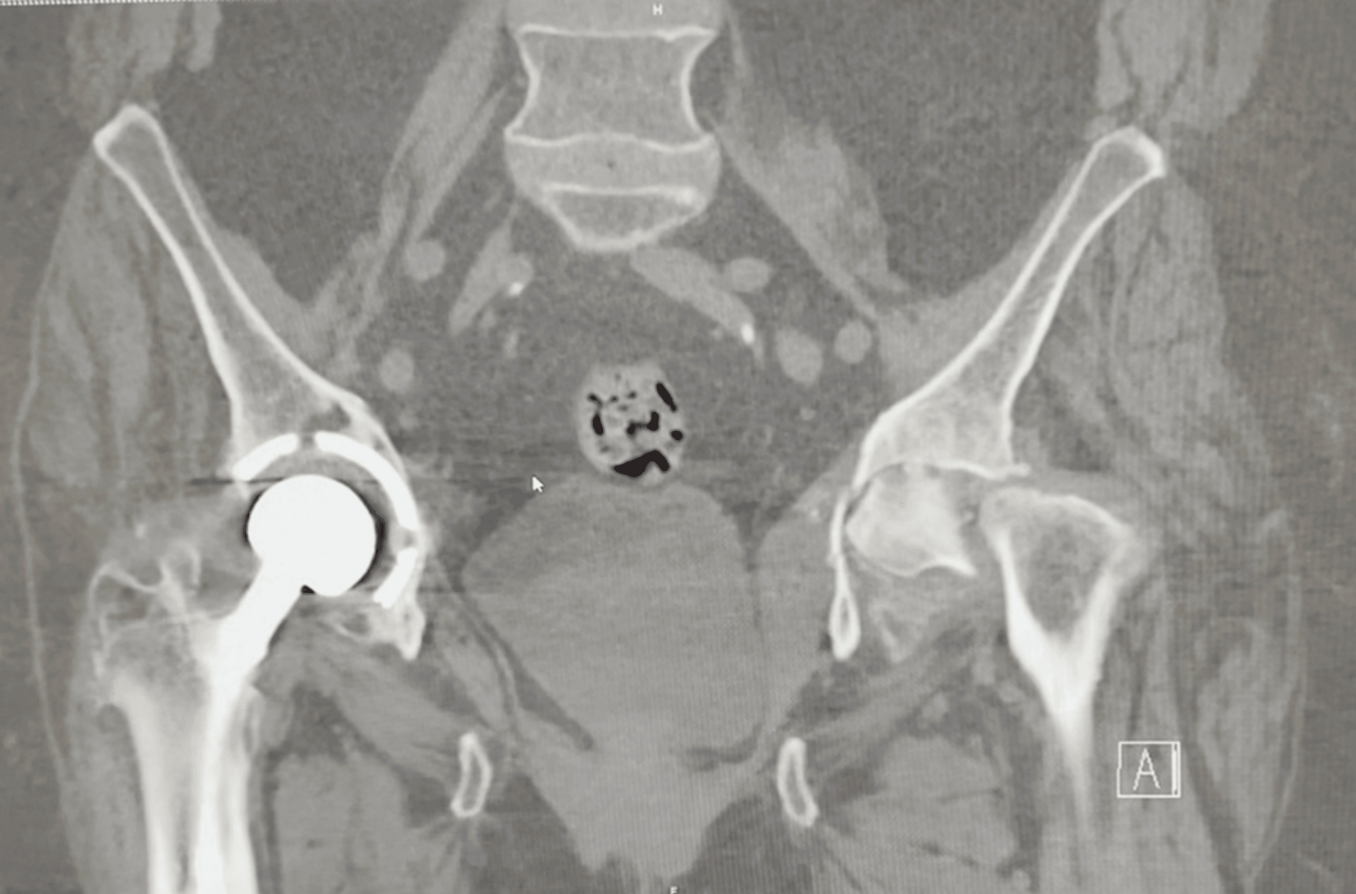
Additional preoperative coronal CT of the pelvis

**Figure 4:**
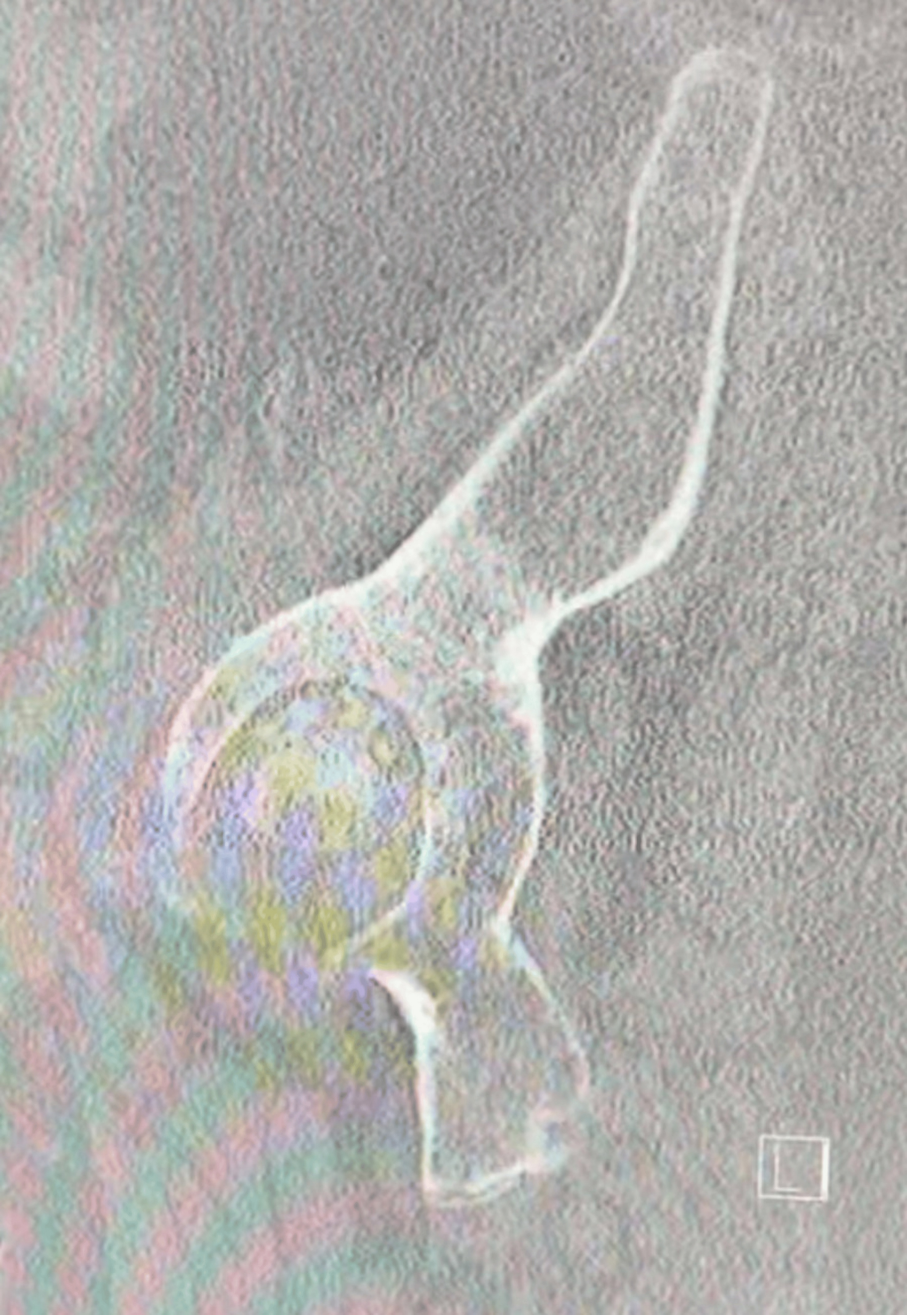
Preoperative sagittal CT of the hip

**Figure 5:**
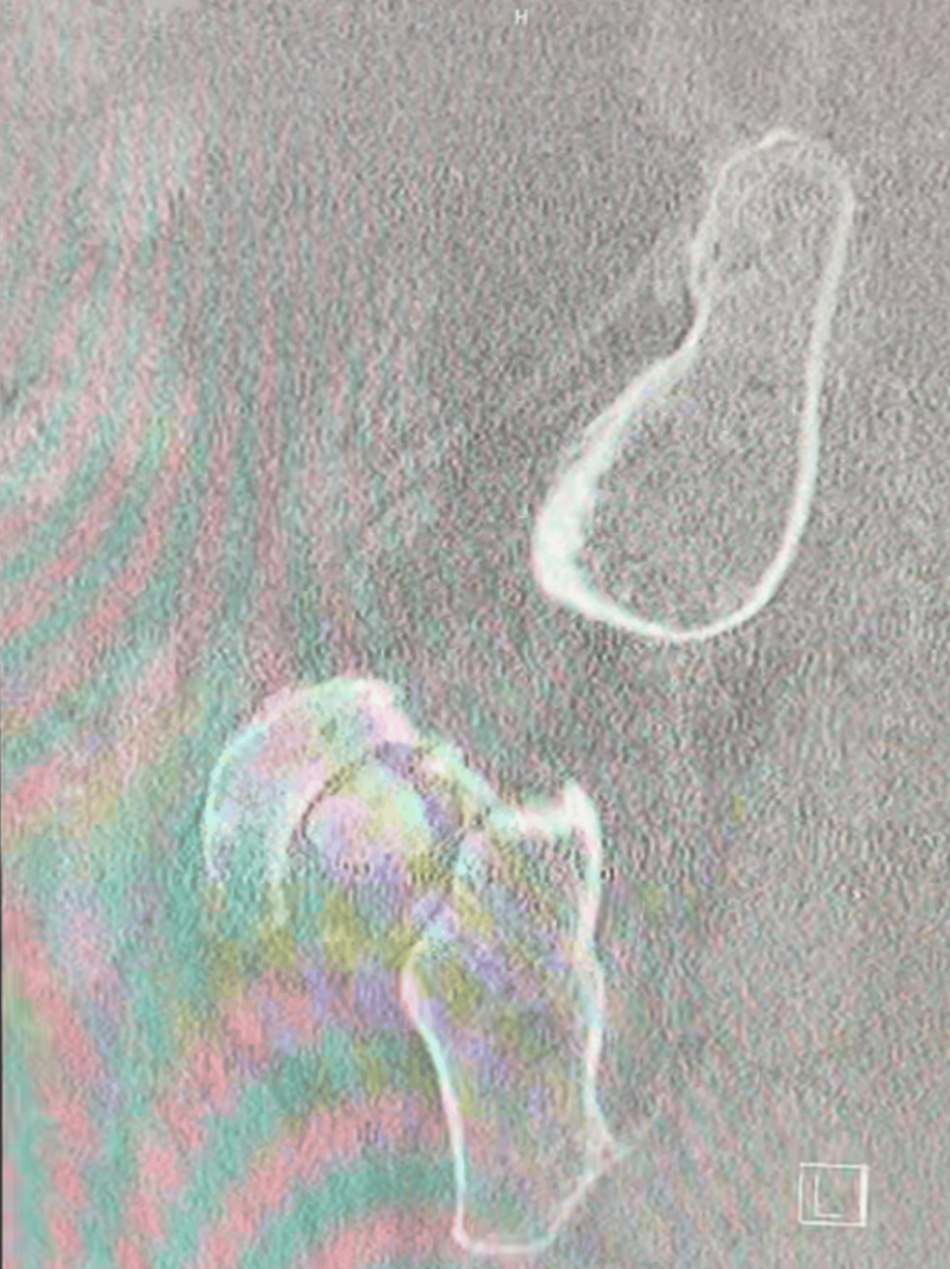
Additional preoperative image of sagittal CT left hip

Imaging revealed a collapse of the left femoral head with associated protrusion (Figure 1). Further evaluation with CT imaging confirmed complete destruction of the left femoral head and progression of acetabular protrusion (Figures 2-5).

The patient was admitted to the inpatient floor for an orthopedic consultation for a fracture of the left femoral head. The CT-guided biopsy demonstrated bone thinning, trabecular microfractures, and femoral head collapse. Additionally, findings were consistent with avascular necrosis and marrow adiposity. Notably, there was no evidence of osteomyelitis or metastatic involvement. Based on the findings from the physical examination and imaging studies, a diagnosis of a pathologic hip fracture secondary to steroid use was made. Other potential diagnoses, such as a displaced medial wall of the left acetabulum and intrapelvic protrusion of the left acetabulum, were also considered.

**Figure 6:**
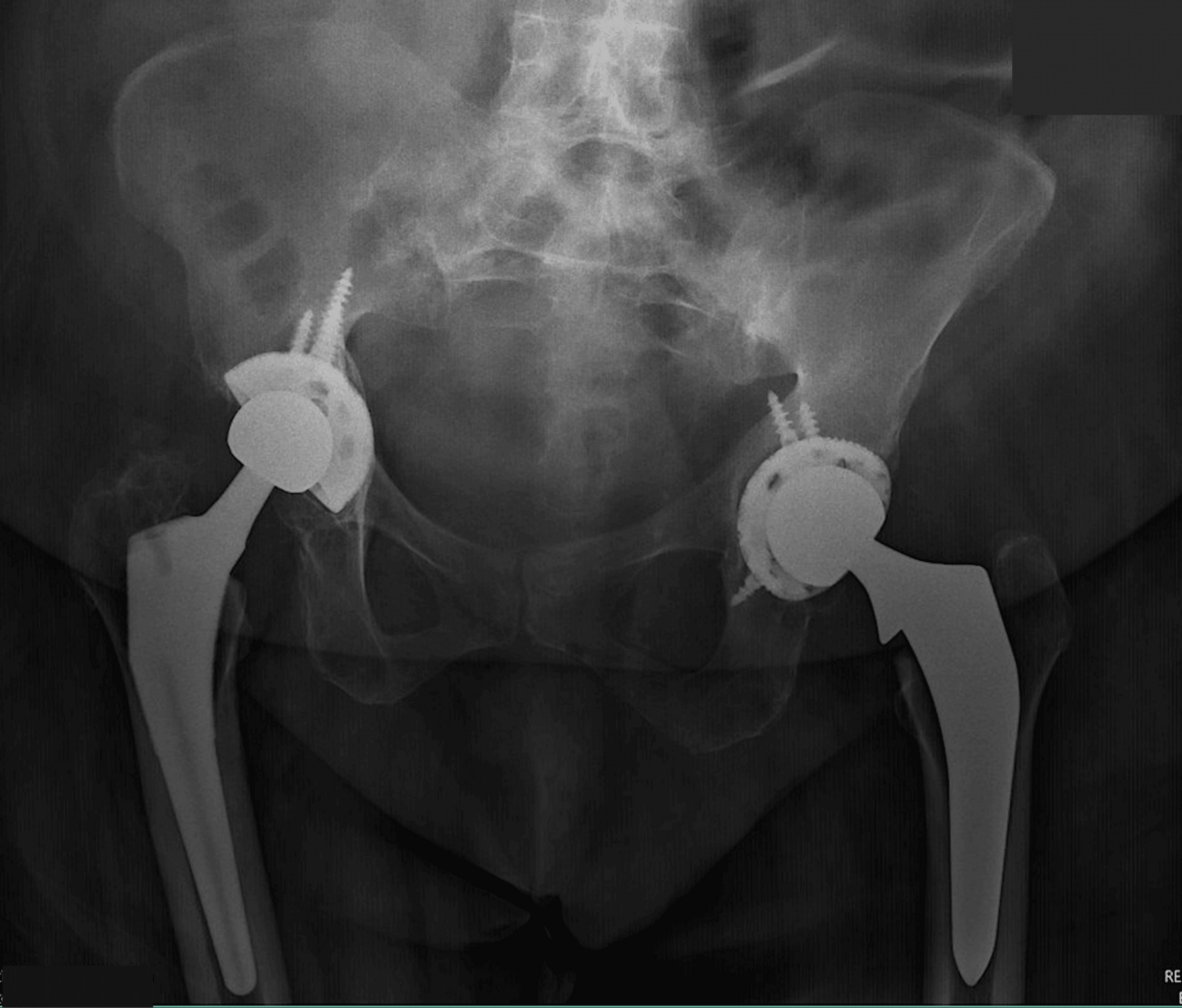
Postoperative AP pelvis AP: anteroposterior

A left total hip arthroplasty was performed. The patient was successfully discharged on postoperative day three and was instructed to follow up at the clinic in 10 days. At the 10-day follow-up, the patient reported minimal pain and was not using any medication for pain management. The postoperative film is displayed in Figure 6.

Verbal consent was obtained from the patient to document the case report regarding their unique presentation of a femoral head fracture.

## Discussion

This case of complete femoral head collapse is unique because of the severity of the fracture after a relatively low dose of steroids. Previous case reports in the literature have identified fractures after corticosteroid use, especially at higher doses. One case demonstrated sclerosis of the femoral head in a 36-year-old man on prolonged steroids [9], although this patient seemed to show a Ficat stage III due to the presence appearing on the X-ray.

The fracture in this case report was mild. Additionally, this patient was on a longer duration and much higher dose of steroids [9]. In another case report, a 65-year-old man experienced bilateral femoral head fracture due to extended steroid use [10]. This patient, however, presented with both a neck fracture and femoral head involvement, and although there was the presence of some femoral head collapse, the severity did not reach Ficat stage IV, as observed in our patient [10]. The Ficat classification is summarized in Table 2.

**Table 2:** Ficat classification

Stage No.	Features	Findings
Stage 0	Normal	Normal
Stage I	Normal or slight osteopenia	Edema
Stage II	Mixed osteopenia, sclerosis, subchondral cysts	Defect seen
Stage III	Crescent sign with some cortical collapse	Same as X-ray
Stage IV	End-stage with visible collapse	Same as X-ray

Mechanism of action

GIOP occurs due to decreased bone formation from suppressed osteoblast activity and increased bone resorption. Biomarkers critical in bone turnover, such as P1NP and osteocalcin, are diminished during glucocorticoid therapy, alluding to impaired bone health [6].

Management strategies

The patient presented with a pathologic hip fracture, highlighting the risks associated with prolonged glucocorticoid use. Lai et al. reported that the risk of fractures increases with a prednisone dose of 5 mg daily for more than three months, regardless of age [3]. The 2022 guidelines recommend using anti-osteoporotic medications, such as bisphosphonates or denosumab, alongside calcium and vitamin D supplementation and regular bone monitoring in at-risk patients [1].

Surgical and postoperative management

Total hip arthroplasty was deemed the appropriate intervention due to the patient's femoral head collapse with acetabular protrusio. Postoperative care included toe-touch weight-bearing precautions, posterior hip precautions, and the use of an abduction pillow. Pain management was achieved with PO acetaminophen and IV hydromorphone for breakthrough pain. The patient was started on standard postoperative antibiotics, receiving cefazolin every eight hours for 48 hours. On postoperative day three, they were instructed to resume their home dose of apixaban, 2.5 mg. A Prevena wound vac and dressing were applied and scheduled for removal 14 days postoperatively during a clinic visit.

Prevention

This case highlights the importance of early assessment of patients undergoing extended glucocorticoid therapy. An assessment could be done through a routine DEXA (dual-energy X-ray absorptiometry) scan starting at age 65. The patient in this case had no record of undergoing a DEXA scan at age 65; the results of this scan may influence a physician's prescription of the corticosteroids. Although the patient's regimen was short in duration, the fracture she sustained underscores the heightened risk of GIOP in not just the elderly but in patients of all ages. This case report also emphasizes the need for individualized risk assessments, particularly in elderly patients. Some patients require consideration of prophylactic measures even for short-term, high-dose steroid use.

## Conclusions

This case highlights the critical impact of glucocorticoid therapy on bone health and the necessity of monitoring and preventive strategies to mitigate GIOP-related risks. Adherence to updated guidelines may reduce fracture risks and improve outcomes, particularly for populations requiring glucocorticoid therapy.
